# Supplementary material for: Increased Ruminoreticular Temperature and Body Activity after Foot-and-Mouth Vaccination in Pregnant Hanwoo (Bos taurus coreanae) Cows
Source: Vaccines (Basel). 2021 Oct 22;9(11):1227. doi: 10.3390/vaccines9111227 (PMC8624786; doi:10.3390/vaccines9111227)
Supplement: Supplementary file 1 [file vaccines-09-01227-s001.zip › vaccines-1379680-supplementary.pdf]

[Supplementary Figures]

A total of 5 adverse events (4 abortion and 1 premature birth) occurred after FMD vaccination. The changes of ruminoreticular temperature and body activity for 5 adverse events are shown in Supplementary figures S1 to S5.

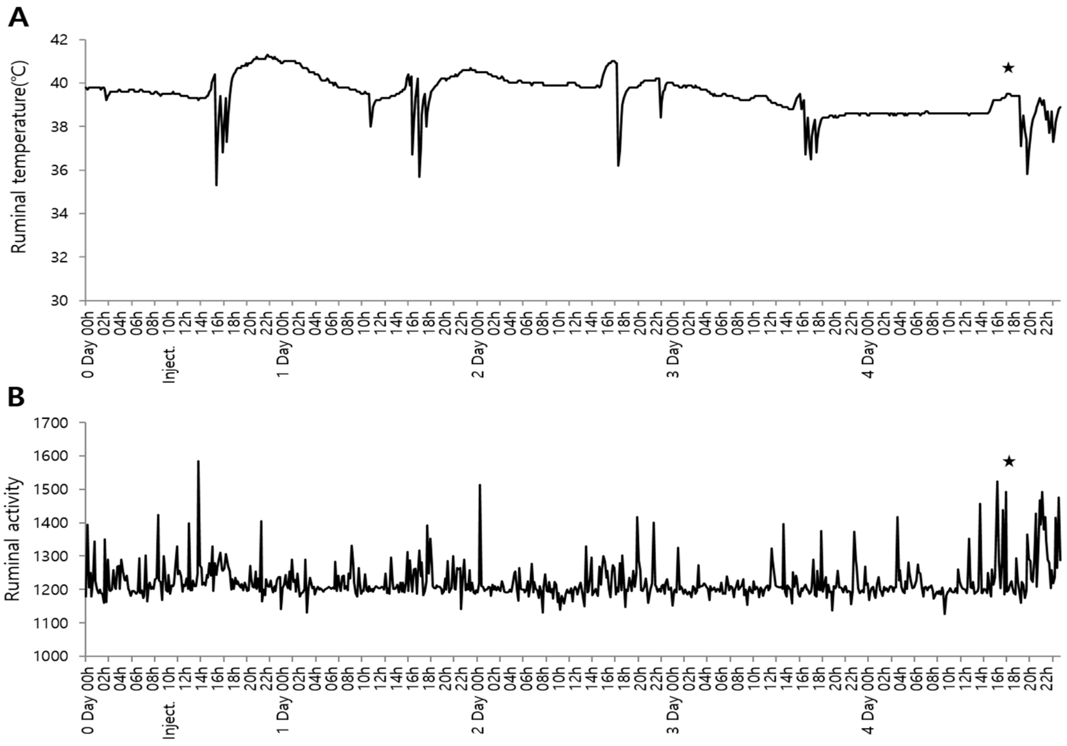

Figure S1. Ruminoreticular temperature and activity in the case of premature birth (#13-81, 274th day of pregnancy). (A) The black line represents ruminoreticular temperature at each 1-h interval. (B) The black line represents body activity at each 1-h interval. "Inject" represents the time of FMD vaccination. The symbol (★) indicates the time of premature birth.

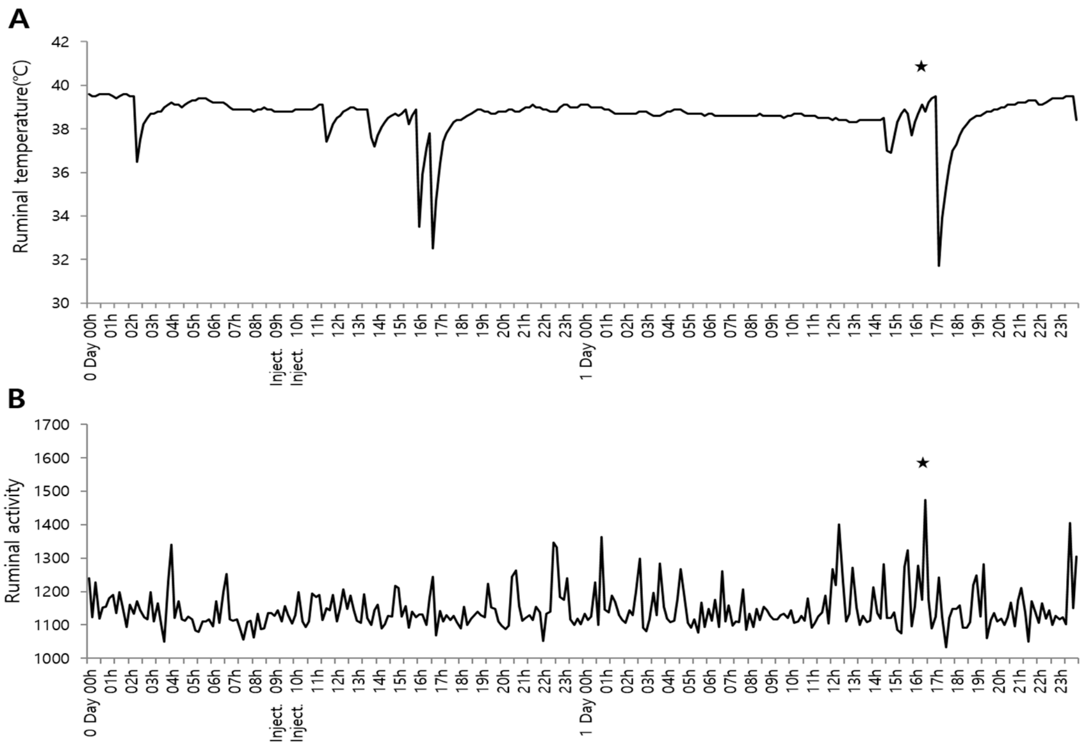

Figure S2. Ruminoreticular temperature and activity in a case of abortion (#433, 267th day of pregnancy). (A) The black line represents ruminoreticular temperature at each 1-h interval. (B) The black line represents body activity at each 1-h interval. “Inject” represents the time of FMD vaccination. The symbol (★) indicates the time of abortion.

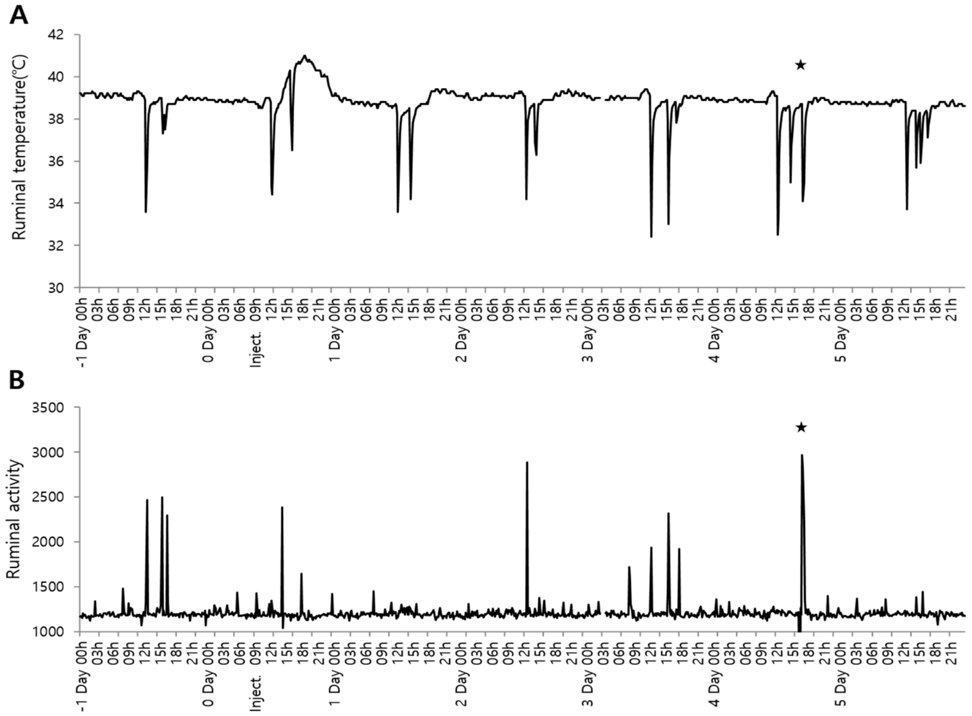

Figure S3. Ruminoreticular temperature and activity in a case of abortion (#17-25, 210th day of pregnancy). (A) The black line represents ruminoreticular temperature at each 1-h interval. (B) The black line represents body activity at each 1-h interval. “Inject” represents the time of FMD vaccination. The symbol (★) indicates the time of abortion.

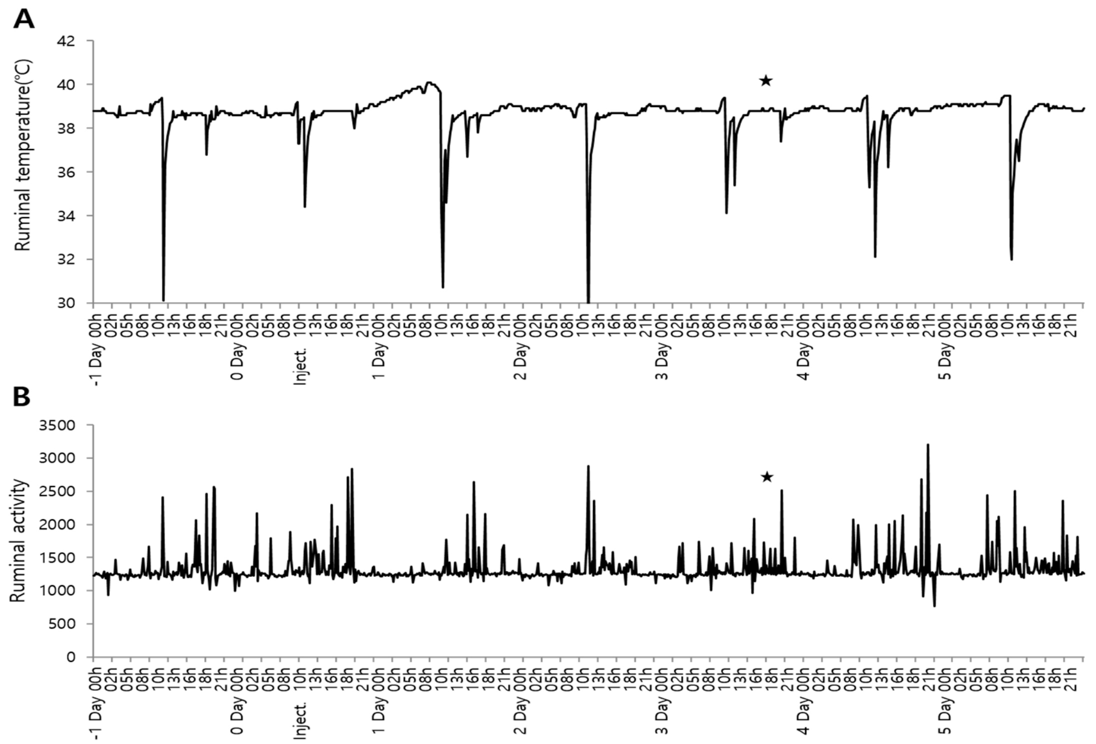

Figure S4. Ruminoreticular temperature and activity in a case of abortion (#425, 68th day of pregnancy). (A) The black line represents ruminoreticular temperature at each 1-h interval. (B) The black line represents body activity at each 1-h interval. "Inject" represents the time of FMD vaccination. The symbol (★) indicates the time of abortion.

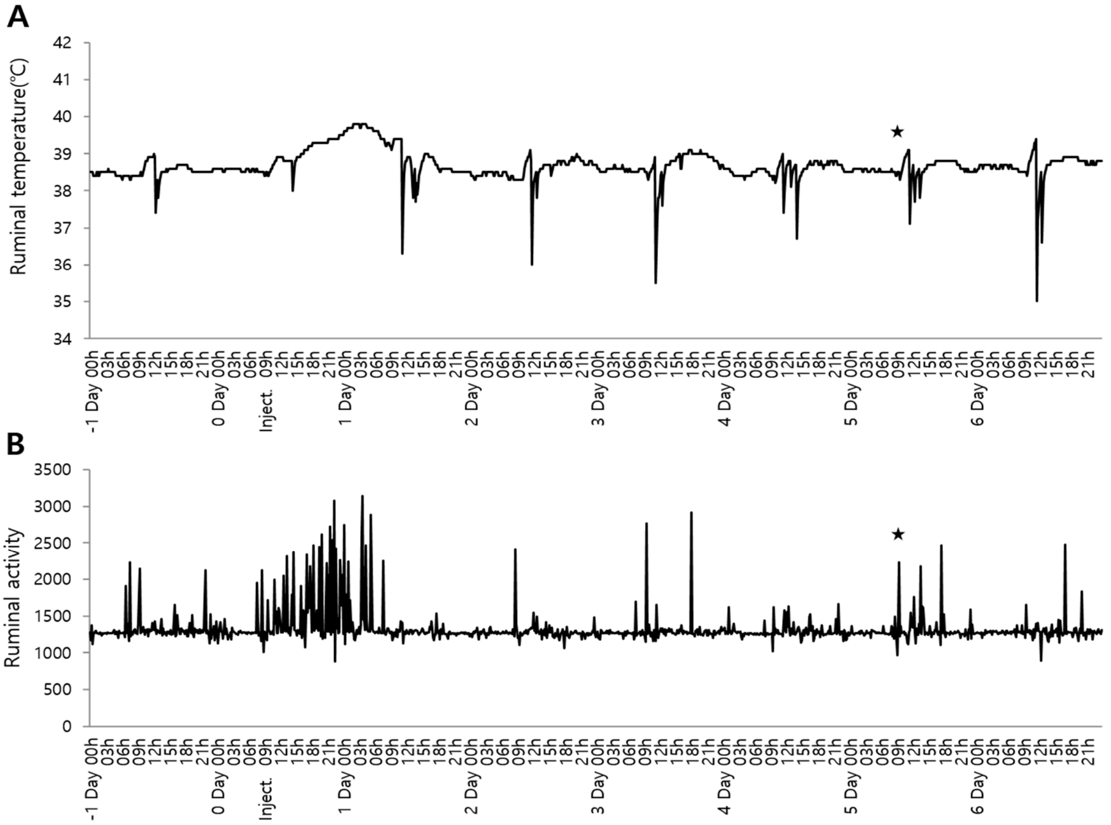

Figure S5. Ruminoreticular temperature and activity in a case of abortion (#398, 47th day of pregnancy). (A) The black line represents ruminoreticular

temperature at each 1-h interval. (B) The black line represents body activity at each 1-h interval. "Inject" represents the time of FMD vaccination. The symbol (★) indicates the time of abortion.
